# Heavy metal content and potential ecological risk assessment of sediments from Khnifiss Lagoon National Park (Morocco)

**DOI:** 10.1007/s10661-022-10002-1

**Published:** 2022-04-11

**Authors:** Ali Tnoumi, Massimo Angelone, Giovanna Armiento, Raffaela Caprioli, Cinzia Crovato, Maurizio De Cassan, Maria Rita Montereali, Elisa Nardi, Luisa Parrella, Marco Proposito, Antonio Schirone, Fabio Spaziani, Bendahhou Zourarah

**Affiliations:** 1 Laboratory of Marine Geosciences and Soil Sciences (URAC 45), Department of Geology, Faculty of Sciences, Chouaib Doukkali University, 24000 El Jadida, Morocco; 2 Casaccia Research Centre, Department for Sustainability, ENEA, 00123 Rome, Italy; 3 Portici Research Centre, Department for Sustainability, ENEA, 80055 Portici, NA Italy; 4 S. Teresa Research Centre, Department for Sustainability, ENEA, 19032 Pozzuolo di Lerici, SP Italy; 5 Present address: ISPRA – The Italian National Institute for Environmental Protection and Research, Rome, Italy

**Keywords:** Heavy metals, Environmental risk, Khnifiss lagoon, Anthropic influence, Geochronological dating, Lagoon sediments

## Abstract

Coastal lagoons are important but sensitive environments, being transitional zones between land and sea. The Khnifiss lagoon is the most important desert wetland in Morocco, but little data have been produced concerning heavy metal geochemistry and enrichments in the sediments. Therefore, 26 surface sediments (15 intertidal and 11 subtidal) and 2 sediment cores were collected in 2016 and analyzed for a selection of heavy metals. The data were processed to assess the degree of contamination and the corresponding potential ecological risk, using several accumulation/enrichment indices, and the singular and multi-metal risk indices. Mean concentrations in the bottom layers of the two cores, dating from a pre-industrial age according to geochronological analysis, were used as the local geochemical background. The resulting values were on the whole lower than those reported for other areas of the northeastern coast of Morocco. Multivariate statistics were also applied to better understand relationships among variables (metals and other geochemical parameters) and to reveal similarities among sample groups. The results showed that, although the lagoon is not yet affected by significant anthropogenic influences, small enrichments can be recognized, especially for Ni and Cd. The cause may be related to the proximity to the main national highway, the vehicles and machinery used in the saltworks located in the area, and the small harbors used principally for fishing. In addition, industrial emissions from the Atlantic coast of Morocco and adjacent countries can be reasonably attributed as additional contributors to the enrichments. In terms of potential ecological risk, Cd shows the greatest impact compared to the other metals investigated.

**Supplementary Information:**

The online version contains supplementary material available at 10.1007/s10661-022-10002-1.

## Introduction

Environmental studies on coastal lagoons and marine estuaries are of considerable importance because these environments are characterized by complex biogeochemical processes. In particular, sediments can become sinks for pollutants (including heavy metals and pesticides), and play a key role in contaminant remobilization in aquatic systems under favorable conditions (Navarrete-Rodríguez et al., 2020).

Trace elements, such as heavy metals, are naturally present in the environment since their origin is closely related to the local geological features. However, in recent decades, many types of anthropogenic activities, such as the use of gasoline that caused the release of high amounts of lead (Pb) in the second half of the last century, have become a significant source of heavy metal emissions. Even remote or protected areas, although not directly affected by anthropogenic emissions (no or little vehicular traffic, industrial activity, tourism, etc.) can be subject to enrichment, since heavy metals are also involved in long-range transport through particulate matter (PM10 and PM2.5) or aerosols (Popoola et al., 2018). Moreover, heavy metal contamination of coastal environments can affect the health of both aquatic organisms and humans (Mountouris et al., 2002), so monitoring activities should be conducted to preserve the original environmental status as much as possible (Mountouris et al., 2002).

Given that element content in sediments is variable depending on the parent material and land use, and not only as a consequence of anthropogenic inputs, the definition of site-specific background levels of relevant elements is essential to assess sediment contamination (Armiento et al., 2011).

Metal content from dated sediments reveal pre-anthropogenic contents and can be effectively used as site-specific reference background values. For aquatic sediments, the use of ^210^Pb originating from the decay of atmospheric ^222^Rn is a well-established methodology to estimate sediment ages and sedimentation rates (Kirchner, 2011).

In this paper, a geochemical investigation of sediments from Khnifiss Lagoon National Park was performed to evaluate the distribution of heavy metal content and anthropogenic contributions, and to estimate the potential ecological risk assessment. Since studies concerning heavy metal contamination in the region are still limited (Idardare et al., 2013; Tnoumi et al., 2021), this paper aims to contribute to improving the geochemical knowledge of the area.

## Geoenvironmental setting

**Fig. 1:**
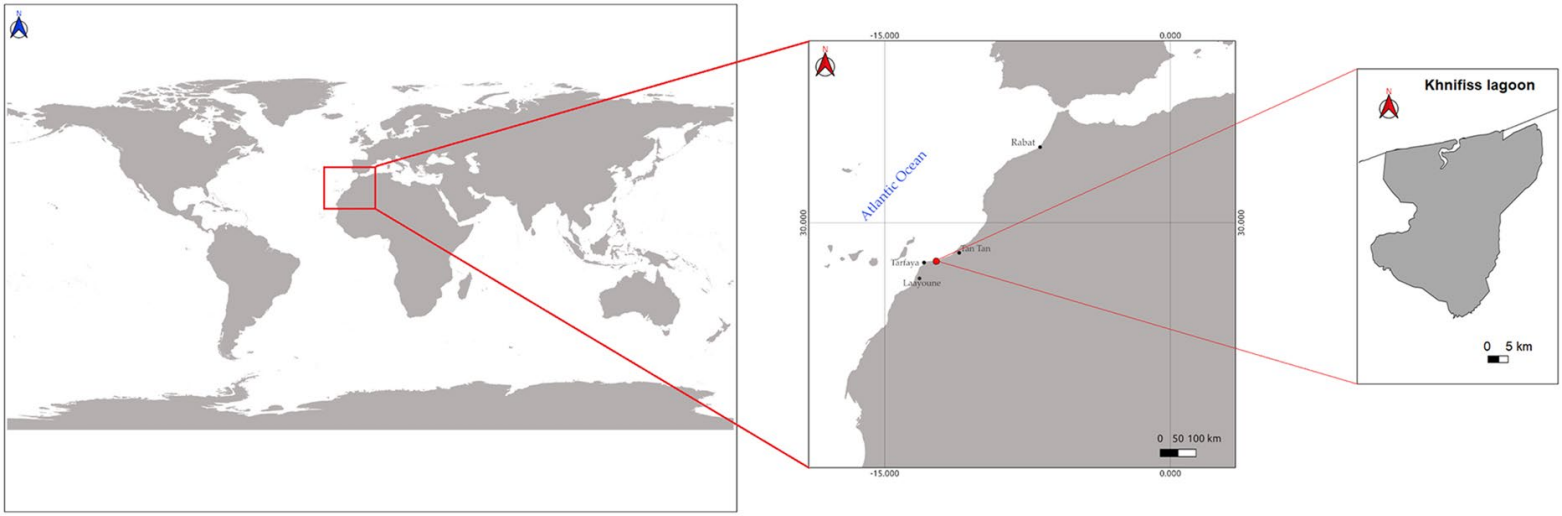
Location of the Khnifiss lagoon

The Khnifiss lagoon (65 km^2^) is the most important desert wetland in Morocco and North Africa, located on the Atlantic Saharan coast (Fig. 1), in the SW of the country, approximately 120 km from the city of Tan-Tan and 70 km from Cape Juby (province of Tarfaya, region of Laayoune-Sakia El Hamra), within the Khnifiss National Park (a protected site and tourist destination of about 1850 km^2^). The area has been designated as a Ramsar site (area of relevance under the “Ramsar Convention on Wetlands of International Importance”) since 1980 and is part of the national network of protected areas as a SIBE site, Site d’Intérêts Biologique et Écologique (Benabid, 2000). To the south of Tan-Tan, an almost flat tabular surface develops, with a general slope on the order of 1% that gradually increases from west to east (Amimi et al., 2017). This extensive plateau, which expands over about 57% of the entire area, consists of a hard calcarenitic plate belonging to the Moghrébien (Upper Pliocene–Lower Pleistocene), which protects underlying marly-calcareous formations of Cretaceous age (Choubert et al., 1966). The area is also particularly relevant from an ecological point of view as it is a wintering site for birds during their migration.

**Fig. 2:**
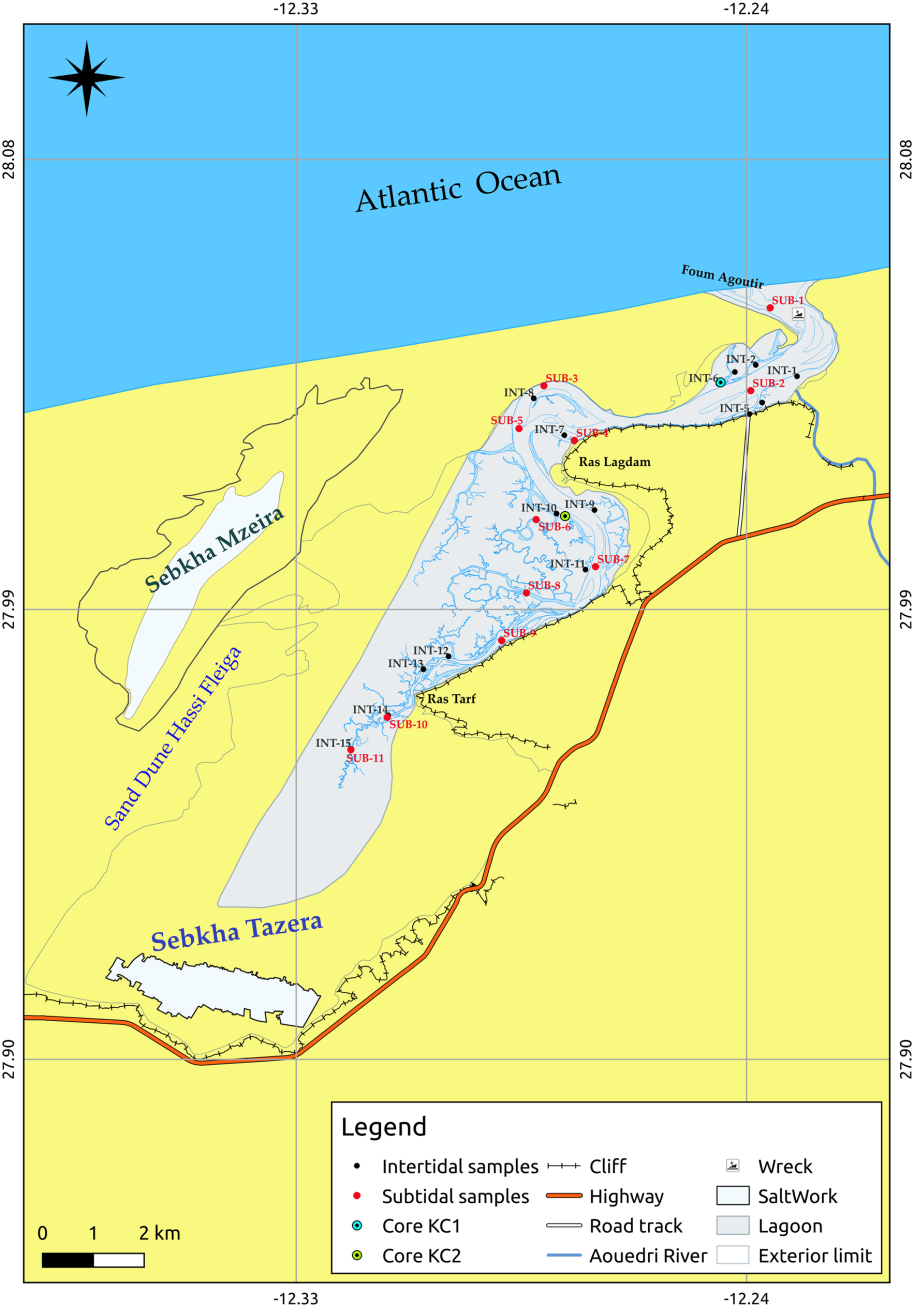
Location of intertidal and subtidal sediment samples in Khnifiss lagoon

The lagoon is characterized by an average tidal range of about 2 m and receives freshwater from occasional rainfall and temporary underground and surface flows from the nearby Oued Aouedri river. The lagoon area consists of a large depression that extends for about 20 km within the coastline and is connected to the Atlantic Ocean by a large lagoon mouth named Foum Agoutir (Fig. 2). It is made of carbonate-based sediments deposited during the Upper Cretaceous (through marine transgressions) and Quaternary deposits of the coastal shelf. These deposits are separated from the Hamada Plateau (on the eastern and southern sides of the lagoon) by a rocky ridge, with an average elevation of 25 m, consisting of sandstones and conglomerates; in the western part and at the outlet, the lagoon is characterized by an impressive system of dunes (Dakki & Parker, 1988; El Agbani et al., 1988; Lakhdar Idrissi et al., 2004; Parker, 1988).

## Materials and methods

### Sampling and analysis

A total of 26 surface sediments were collected, as individual samples, during spring of 2016 (Fig. 2): 15 samples (from INT1 to INT15) were intertidal sediments, collected from the area that is submerged during high-tide periods; 11 samples (from SUB1 to SUB11) were subtidal sediments, sampled from the area that is always underwater. Samples were collected from a 0- to 5-cm depth using a stainless steel Van Veen grab sampler. Each sample was carefully taken from the central portion of the collected sediment with a plastic spatula previously washed with lagoon water, and then placed in pre-cleaned polyethylene bags. Two cores (KC1, from the downstream, and KC2, from the midstream of the lagoon) were also sampled to define the local background, by introducing a 50-mm-diameter PVC core sampler down to a depth of approximately 100 cm and 300 cm, respectively. The sampling sites were selected to avoid dredged areas and navigating channels, to minimize the risks of collecting disturbed sediments. At the laboratory, the cores were then subdivided into 2-cm subsamples from top to bottom. All samples were refrigerated at 4 °C until transferred to the analytical laboratory.

From each surface sediment sample, two aliquots were separated: one for geochemical analyses (major/trace element quantifications and particle size) and one for organic matter analysis. Aliquots for geochemical analyses were oven-dried at 40 °C, while those for organic matter determination were freeze-dried.

The oven-dried aliquots were sieved using a mechanical sieving device to obtain the fraction below 2 mm, which was used for geochemical analyses. Particle size analysis was performed using both dry and wet sieving techniques: samples were wet-separated into coarse and fine fractions using a 63-μm mesh sieve. The coarse fraction was dried again and then separated using ASTM series sieves. The wet fine fraction was analyzed using a SediGraph 5100 (Micromeritics) after being dispersed in a 0.5% sodium hexametaphosphate solution. Geochemical characterization was performed using a microwave-assisted acid digestion procedure according to EPA Method 3052, followed by ICP-MS analysis (PerkinElmer ELAN 6100), for trace elements (As, Cd, Co, Cr, Cu, Ni, Pb, V, Zn), and ICP-OES (PerkinElmer OPTIMA-2000 DV), for major elements (Al, Ca, Fe, K, Mg, Mn, Na, Ti). The quality of the analytical data was assessed using procedural blanks and by analyzing, with the same method adopted for the samples, two certified reference materials based on marine sediment (PACS-3 and MESS-4, National Research Council Canada, NRCC). Recoveries of Al, As, Cd, Co, Cr, Cu, Ni, Pb, V, and Zn ranged from 84 to 119% for MESS-4 and from 88 to 101% for PACS-3. Limits of quantitation (*LOQ*s) were as follows: As, Co, Pb, and V = 0.05 mg/kg; Cd = 0.03 mg/kg; Cu and Ni = 0.3 mg/kg; Cr = 0.5 mg/kg; Mn and Zn = 1 mg/kg; Fe = 5 mg/kg; Ca, Mg, and Ti = 10 mg/kg; Na = 20 mg/kg; and Al and K = 50 mg/kg (Tnoumi et al., 2021). Organic matter (OM), which in lagoon sediments comes from both marine and terrestrial sources, was determined by the loss on ignition (LOI) procedure (Heiri et al., 2001).

Radiometric analyses were also performed by gamma spectrometry. Each sample was counted for 2 days on high-purity germanium detectors to determine ^210^Pb, ^226^Ra, and ^137^Cs activities. The excess ^210^Pb activity (^210^Pb_ex_) was calculated as the difference between the total ^210^Pb and ^226^Ra. Details on calibrations, quality checks, and measurements have been presented elsewhere (Delbono et al., 2016). The ^210^Pb dating method has been widely used to establish geochronology in marine sediments on timescales of ~110 years (Sanchez-Cabeza & Ruiz-Fernández, 2012, and references therein). This method is based on the decay of the unsupported ^210^Pb_ex_ fraction present in the sediment and must always be verified using an independent temporal marker (Smith, 2001). For this purpose, ^137^Cs is frequently used; this artificial radionuclide was introduced into the environment primarily during atmospheric nuclear weapon testing (NWT) until 1963, when these tests were banned. To achieve dating, the constant rate of supply (CRS) model (Appleby & Oldfield, 1978; Robbins et al., 1977) was applied to the KC1 sediment core, assuming a constant flux of ^210^Pb_ex_ at the sediment–water interface and a variable mass accumulation rate (*MAR*, g/(cm^2^ year)). Due to irregularities in deposition, proper dating could not be established in core KC2.

### Data elaboration

Several data elaboration techniques were used to estimate metal pollution in sediments, including general and multivariate statistics. In addition, a selection of indices was applied to assess the degree of contamination and the corresponding ecological risk.

#### Background matrix

To properly interpret geochemical data, selecting a background matrix is an essential step in highlighting enrichments/dissimilarities. Commonly used background matrices are the “average shale” or “average upper continental crust,” but the availability of a local background is undoubtedly recommended and should be employed. The selection of the most appropriate matrix is a critical issue since discordant results and assessments can be obtained utilizing different background data. In this work, the two abovementioned local sediment cores were used to define the background matrix; indeed, considering both the heavy metals’ depth-profile results and the chronological dating, some layers from the lower segments of the sediment cores (2 layers from KC1 and 3 layers from KC2), dating back to about 100 years and beyond, were evaluated as pristine and the element averages were used as the local geochemical background (*LGB*).

#### Data normalization

Before the data processing and interpretation using multivariate statistics, normalization was applied. This preliminary step is necessary because metals in sediments can derive from both natural sources (such as bedrock material) and anthropogenic sources. Therefore, to assess the degree of contamination, it is important to differentiate the actual contribution from these origins. Input from natural sources could differ by several orders of magnitude, depending on mineralogy and particle size distribution (Loring, 1991), and could lead to an overestimation of the anthropogenic contribution. Consequently, a normalization procedure should be used to compensate for particle size and mineralogical effects on metal content.

A typical geochemical approach is the normalization of metal data based on the content of a conservative element (Loring, 1990; Schropp & Windom, 1988). Among the parameters in our database, Al, Fe, and Ti showed very good correlations with the other elements (both major and trace) and could be used as normalizers. However, Al exhibited the best correlation (*r* = 0.87) with the fine fraction (silt + clay), and also high correlations with all heavy metals (*r* values ranging from 0.68 to 0.89). Therefore, it was judged to be the most appropriate for the Khnifiss lagoon sediments.

#### Assessment of sediment enrichment and potential ecological effects

The degree of heavy metal enrichment in the surface sediments, and the related potential ecological effects, were estimated by a selection of indices (Table 1) calculated using the abovementioned *LGB* as background values: the enrichment factor (*EF*) (Grant & Middleton, 1990; Salomons & Forstner, 1984; Woitke et al., 2003), the geoaccumulation index (*Igeo*) (Muller, 1969; Ntekim et al., 1993), the pollution load index (*PLI*) (Tomlinson et al., 1980), the modified degree of contamination (*mCd*) (Abrahim & Parker, 2008), and the risk index (*RI*) (Hakanson, 1980).

**Table 1:** Indices used to assess the degree of heavy metal enrichments and the potential ecological effects

Index	Formula	Reference
Enrichment factor (*EF*)	$EF={(M/RE)}_{\mathrm{s}}/{(M/RE)}_{\mathrm{b}})$ (*M*/*RE*) is the concentration ratio of a metal and a reference element (Al) in the sample (s) and in the background matrix (b)	Salomons and Forstner (1984), Grant and Middleton (1990), Woitke et al. (2003)
Geoaccumulation index (*Igeo*)	$Igeo={\mathrm{log}}_{2}(Cn/1.5\times Bn)$ *Cn* and *Bn* are the concentrations of a metal in the sample and in the background matrix	Muller (1969), Ntekim et al. (1993)
Contamination factor (*CF*)	$CF={Cm}_{\mathrm{s}}/{C}_{\mathrm{b}}$ *Cm*_s_ is the metal concentration in the sample, and *C*_b_ is the metal concentration in the background matrix	Hakanson (1980)
Modified degree of contamination (*mCd*)	$mCd={\sum }_{i=1}^{i=n}CF/n$ *n* is the total number of the metals considered	Abrahim and Parker (2008)
Pollution load index (*PLI*)	$PLI=\sqrt[n]{{CF}_{1}\times {CF}_{2}\times \dots {CF}_{n}}$ *n* is the total number of the metals considered	Tomlinson et al. (1980)
Ecological risk indices (*Er* and *RI*)	$RI=\sum\limits_{i=1}^{n}{Er}_{i}=\sum\limits_{i=1}^{n}{T}_{i}\times \mu {CF}_{i}$ *T* is the toxicity coefficient for a given element as follows: As = 10, Cd = 30, Co = 5, Cr = 2, Cu = 5, Mn = 1, Ni = 5, Pb = 5, V = 2, Zn = 1 *µCF* is the mean value of the contamination factors for a given element *n* is the total number of the elements considered	Hakanson (1980)

## Results and discussion

### Core geochronological dating

**Fig. 3:**
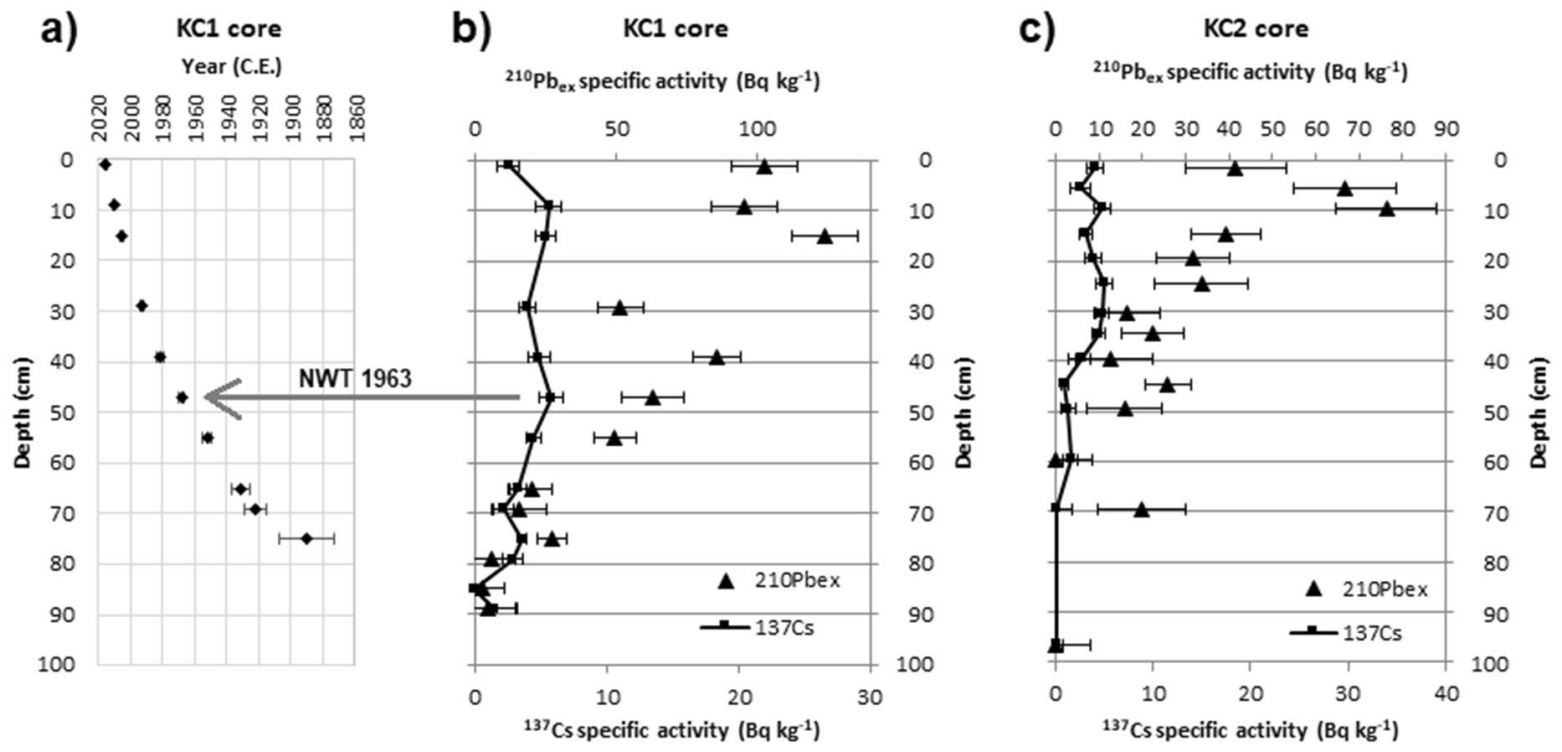
Correspondence between layer depth and age in the KC1 sediment core (**a**); ^210^Pb_ex_ and ^137^Cs vertical profiles in KC1 (**b**) and KC2 (**c**) sediment cores. The arrow marks the maximum ^137^Cs activity

In the KC1 sediment core, the CRS age model allowed estimation of the age of the layers up to the equilibrium depth, where ^210^Pb_ex_ falls to zero (Fig. 3a). The vertical profile of ^210^Pb_ex_ (Fig. 3b) did not show regular exponential decay, thus confirming that constant sedimentation rate could not be assumed. The deep maximum of ^137^Cs was observed in the 46–48-cm layer, in agreement with the calculated age (1968 ± 2). Considering that not all layers were analyzed, this can be considered the required confirmation of ^210^Pb dating. It should be noted, however, that ^137^Cs did not disappear in the layers older than 1954, and this is a feature related to the fraction of ^137^Cs that is diffuse in interstitial water (Abril et al., 1991). The CRS model also allowed *MAR* to be estimated for each layer: in this core, *MAR* was higher in layers younger than 1968 (mean value = 1.2 ± 0.4 g/(cm^2^ year) compared with 0.5 ± 0.2 g/(cm^2^ year) in the older layers), comparable with values in other lagoons in Morocco (Benmhammed et al., 2021; Maanan et al., 2013, 2014).

In the KC2 sediment core, the ^210^Pb_ex_ and ^137^Cs profiles did not yield a confirmed dating. However, it was possible to conclude that the layers in which ^210^Pb_ex_ and ^137^Cs are negligible (deeper than 70 cm) had no relevant input from the surface during the last 110 years (Fig. 3c).

The use of sediment cores provides not only background levels, but also produces a record of contamination and when dated, as in this case, establishes the age of onset of pollution (Birch, 2017).

### General statistics

The general statistics of the analysis results are summarized in Table 2 (for intertidal sediments) and Table 3 (for subtidal sediments). The average concentrations of major elements in surface sediments were lower than those reported for the upper continental crust (UCC; Rudnik & Gao, 2014), except for Ca, which showed quite higher amounts in both intertidal and subtidal sediments (Table 4) that may be related to eolian and biogenic inputs (Tnoumi et al., 2021). The distribution of OM was heterogeneous, showing significant variation between intertidal (higher values) and subtidal (lower values) samples. The OM content in sediments has many sources such as terrigenous deposits, marine deposits, and biota decomposition (Bentivegna et al., 2004). However, the state of the lagoon and the absence of terrestrial inputs suggest that marine productivity is the primary source of OM in this area (Tnoumi et al., 2021).

**Table 2:**
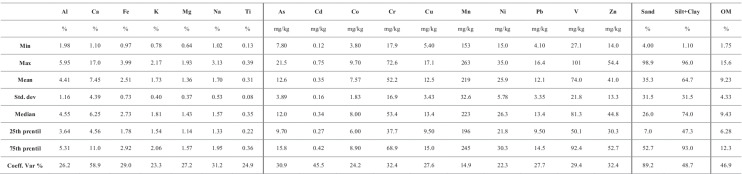
Intertidal sample summary statistics (15 samples)

**Table 3:**
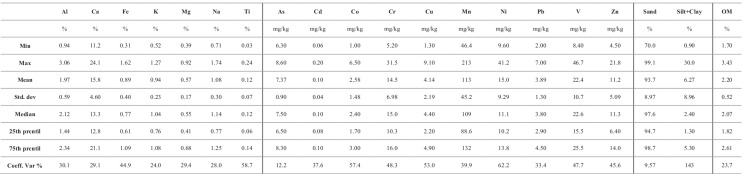
Subtidal sample summary statistics (11 samples)

**Table 4:**
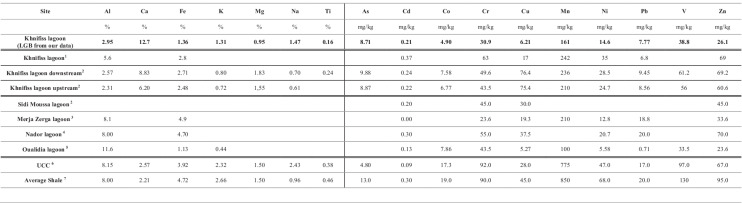
Local geochemical background (*LGB*) of the Khnifiss lagoon calculated from the results of our analyses, and its comparison with literature concerning metal concentrations in intertidal sediment from the same area, local backgrounds of other Moroccan lagoons, and commonly used reference data (UCC and average shale)

^1^Idardare et al. (2013)

^2^Boutahar et al. (2019)

^3^Maanan et al. (2013)

^4^Maanan et al. (2015)

^5^Mejjad et al. (2018)

^6^Rudnik and Gao (2014)

^7^Turekian and Wedepohl (1961)

Heavy metal concentrations in surface sediments showed, on the whole, the following sequences: Mn > V > Cr > Zn > Ni > As > Pb > Cu > Co > Cd (for intertidal samples) and Mn > V > Ni > Cr > Zn > As > Cu > Pb > Co > Cd (for subtidal samples). In addition, for all metals investigated, trends showed decreasing concentration levels as sediment grain size increased. Our results are in excellent agreement with the data produced by Idardare et al. (2013), for intertidal sediment samples from the same area (Table 4).

The coefficient of variation for heavy metals ranged from 14.9 to 45.5% for the intertidal samples and from 12.2 to 62.2% for the subtidal samples. In particular, As, Cd, Cr, and Zn, for intertidal sediments, and all heavy metals except As, for subtidal sediments, were characterized by variation greater than 30%, showing considerable spatial variability. Such outcomes imply that heavy metal occurrences at each site are potentially affected by several factors including human activities.

The sediment cores exhibited decreasing trends from top to bottom in metal content. The bottom layers were datable before the onset of massive anthropogenic inputs (pre-industrial age). This confirmation allowed us to hypothesize that the average element concentrations of the bottom layers can be considered as the *LGB* of the area. The resulting sequence of heavy metal concentrations is Mn > V > Cr > Zn > Ni > As > Pb > Cu > Co > Cd, which is very similar to that obtained for the surface samples. This is a first hint suggesting that the lagoon sediments are not significantly affected by contamination. The *LGB* data for the Khnifiss lagoon are reported in Table 4, where local background values for other Moroccan lagoons acquired from various sources and the aforementioned UCC and average shale are also summarized.

### Multivariate statistics analysis

To evaluate the data in their wholeness, a multivariate statistical approach was applied. Principal component analysis (PCA) and cluster analysis were performed on the complete surface sediment dataset. According to literature, multivariate statistics produces reliable results when executed on a sufficiently large dataset (e.g., Comrey and Lee (1992) suggested that a reasonable size is about 200 samples), or on a dataset with a certain minimum sample to variable ratio (Cattell (1978) recommended a ratio of 3:1). Therefore, being aware that due to the small size of the dataset proposed in this article, and the not optimal cases/variables ratio, multivariate statistics cannot be performed rigorously; the results proposed here must be intended as merely indicative.

Bartlett’s sphericity test and the Kaiser–Meyer–Olkin index (0.001 and 0.72 respectively, *p* < 0.01) indicated that PCA is suitable on this dataset as a reduction tool. The first two PCA components obtained explain about 80% of the entire dataset variance. The first factor (accounting for about 59% of the dataset total variance) is mainly driven by Al, Mg, Ti, Fe, Na, K, OM, fine fraction (silt + clay), and most of the heavy metals. Their significant positive correlations might represent common sources for these metals. The contribution of Al, K, and Fe, in particular, may imply the role of clay minerals and Al and Fe oxy-hydroxides in the adsorption of heavy metals. Moreover, the positive correlation between OM and the abovementioned parameters also suggests the OM contribution in the accumulation of heavy metals in the sediments. Factor 2 (accounting for about 22% of the total variance) is mainly characterized by Mn, As, Cu, Pb, V, Cr, Zn, and Co. These elements showed a moderate mutual correlation, assuming their sources from various local pollution such as (i) vehicular traffic along the nearby highway; (ii) saltworks activities and related heavy machines – the sebkha Tazera contributes with other sebkhas about 20,000 tons/year of salt production; and (iii) boats used for tourism and fishing activities. Pb is a marker of vehicular traffic emissions which contributes more than 50% in the metal’s total release (Pulles et al., 2012). Zn is reasonably associated with the combustion of lubricating oil, and the highest levels have been found near the wood docks and the salt pans, while Cr and Cu are the markers of antifouling paints (Pulles et al., 2012).

**Fig. 4:**
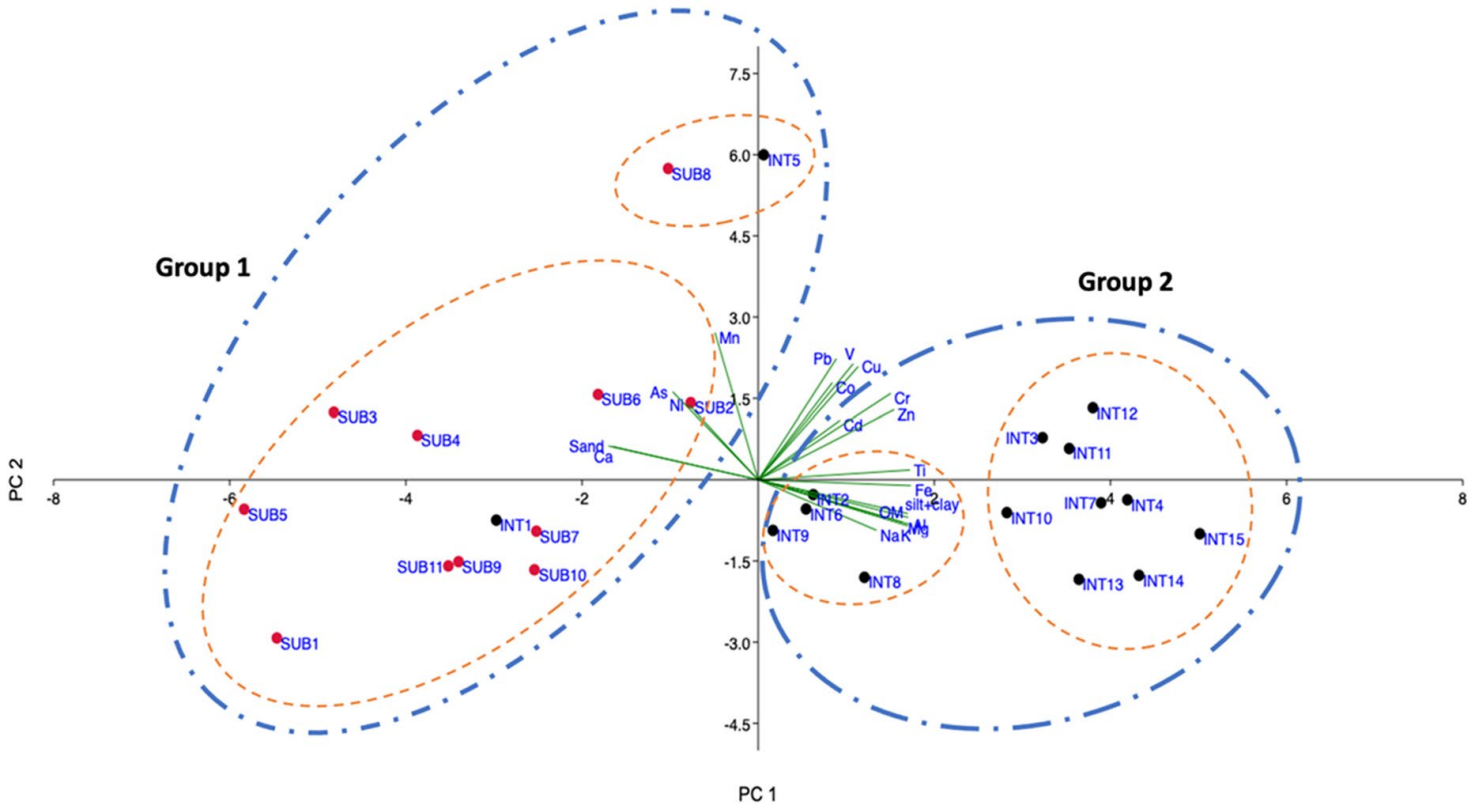
Principal component analysis (PCA) performed on surface sediments of the Khnifiss lagoon

The resulting PCA scatter plot (Fig. 4) showed that the lagoon sediments can be gathered into two groups whose differentiation is mainly related to the amount of OM and fine fraction. Indeed, “group 1” includes the samples characterized by the lowest levels of trace metals, OM, and fine fraction (silt + clay) in the dataset; these parameters are closely related since OM contains functional groups that can form complexes with trace metals, and the fine fraction has a higher surface area and adsorption capacity (Fernandes et al., 2011; Sadeghi et al., 2012). In contrast, “group 2” includes samples with the highest values of trace metals, OM, and fine fraction. Furthermore, each group can be split into two further subcategories characterized by minor differences: the subcategory of group 1 that includes the INT5 and SUB8 samples is particularly interesting since the orientation of the PCA vectors and the substantial distance of the samples from the other subcategory are indications of relevant differentiation in terms of metal enrichment.

The PCA also highlighted the existing geochemical distinction between intertidal and subtidal sediments, as they proved to be well distinguishable in the two groups: indeed, except for samples INT1 and INT5, all intertidal samples were part of group 2, while all subtidal samples were included in group 1.

**Fig. 5:**
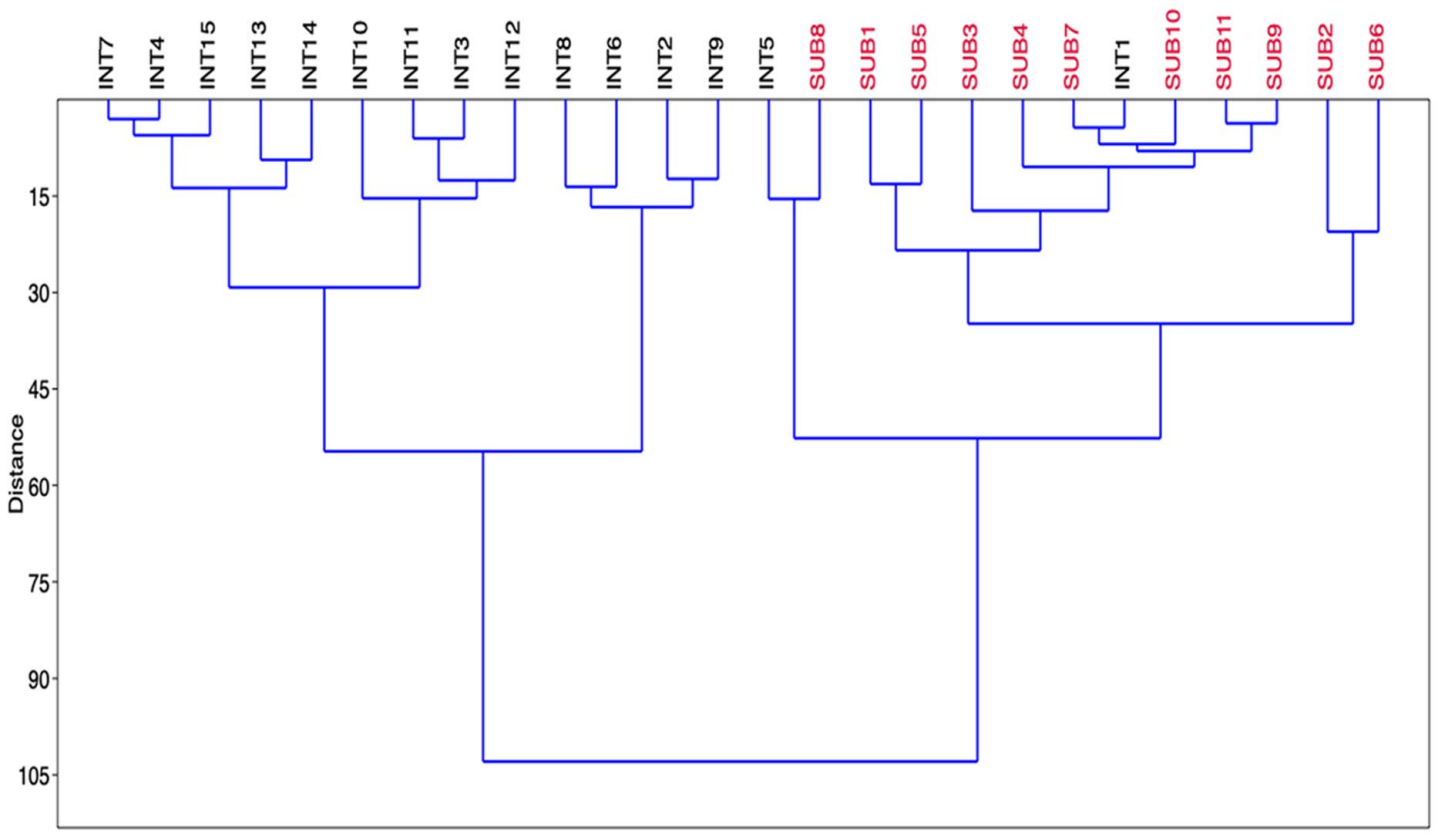
Cluster analysis for the surface sediments of the Khnifiss lagoon

Cluster analysis confirmed the PCA results since the same groups and subgroups are clearly distinguished (Fig. 5).

### Assessment of the enrichment degree

Although multivariate statistics highlighted some samples exhibiting signs of enrichment, and a group of samples with higher trace element concentration levels compared to the others, it is not sufficient to assess the state of the lagoon sediments. Indeed, it is necessary to compare the data with a reference matrix to evaluate whether the presumed metal enrichments are significant or negligible.

#### Enrichment factor (*EF*) results

The results of the *EF* calculations are arranged as electronic supplementary material in Table ESM1. According to the *EF* guideline interpretation proposed by many authors (e.g., Chen et al. (2007)), metal contamination can be classified into the following categories: *EF* ≤ 1, no enrichment; 1 < *EF* ≤ 3, minor enrichment; 3 < *EF* ≤ 5, moderate enrichment; 5 < *EF* ≤ 10, moderately severe enrichment; 10 < *EF* ≤ 25, severe enrichment; 25 < *EF* ≤ 50, very severe enrichment; and *EF* > 50, extremely severe enrichment. The results found on the Khnifiss surface sediments showed no enrichment or evidenced only minor enrichments. The highest values were found for Ni (3.0) in sample SUB3 and Cd (2.6) in sample INT12. The elements that showed the most occurrences of “minor enrichment” were Ni (20 samples), Cu (18 samples), and V (15 samples). Sediments INT5 and SUB8 are those that especially displayed an overall sign of “minor enrichment,” due to the higher incidence of elements having *EF* > 1, and also evidenced by the highest mean *EF* values (1.6 ± 0.5 and 1.6 ± 0.4, respectively); this finding confirms the results of the multivariate statistics since these two samples are the same belonging to the abovementioned subcategory of group 1 in the PCA.

For comparison purposes, we also calculated the *EF*s using the widely adopted “average shale composition” as background values (Turekian & Wedepohl, 1961), and then matched the results with those obtained from *LGB*. As reported in Table 5, the *EF*s determined using the shale data are sensibly higher for As, Cd, and Cr. In particular, for Cr, the values denote a condition of “moderately severe enrichment” for intertidal samples and “moderate enrichment” for subtidal samples (two categories and one category higher than the “minor enrichment” resulting from the *EF*s obtained using the local background, respectively). In contrast, *EF*s for Co, Cu, Mn, Ni, Pb, and Zn are higher when calculated using *LGB*. This comparison underlines the importance of a wise and proper selection of the reference matrix used in the *EF* calculation and the importance of the availability of local background values, to avoid overestimates/underestimates and thus inaccurate classifications that do not reflect the true environmental context.

**Table 5:** Comparison between *EF*s calculated using the local geochemical background and the average shale composition

	**As (mg/kg)**	**Cd (mg/kg)**	**Co (mg/kg)**	**Cr (mg/kg)**	**Cu (mg/kg)**	**Mn (mg/kg)**	**Ni (mg/kg)**	**Pb (mg/kg)**	**V (mg/kg)**	**Zn (mg/kg)**
***EF*** ** (local background) intertidal samples**	1.0 ± 0.3	1.1 ± 0.5	1.0 ± 0.1	1.1 ± 0.2	1.3 ± 0.2	1.0 ± 0.3	1.2 ± 0.2	1.0 ± 0.2	1.3 ± 0.4	1.0 ± 0.1
***EF*** ** (average shale) intertidal samples**	2.1 ± 0.8	2.2 ± 1.0	0.8 ± 0.2	5.1 ± 1.2	0.6 ± 0.1	0.5 ± 0.3	1.1 ± 0.3	0.2 ± 0.1	1.7 ± 0.7	0.8 ± 0.2
***EF*** ** (local background) subtidal samples**	1.5 ± 0.5	0.8 ± 0.2	0.8 ± 0.3	0.8 ± 0.3	1.1 ± 0.4	1.1 ± 0.3	1.7 ± 0.7	0.8 ± 0.2	0.9 ± 0.3	0.7 ± 0.3
***EF*** ** (average shale) subtidal samples**	3.0 ± 1.0	1.7 ± 0.5	0.6 ± 0.3	3.5 ± 1.2	0.4 ± 0.2	0.6 ± 0.2	1.5 ± 0.6	0.1 ± 0.0	1.2 ± 0.4	0.6 ± 0.2

*EF* for each element expressed as the average (mean ± std. dev.) of the *EF*s calculated on the surface sediments (intertidal or subtidal)

#### Geoaccumulation index (*Igeo*) results

The *Igeo* results are collected as electronic information in Table ESM2 and are classified according to the following criteria (commonly acknowledged by many authors, such as Nilin et al. (2013)): *Igeo* ≤ 0, uncontaminated; 0 < *Igeo* ≤ 1, uncontaminated to moderately contaminated; 1 < *Igeo* ≤ 2, moderately contaminated; 2 < *Igeo* ≤ 3, moderately to heavily contaminated; 3 < *Igeo* ≤ 4, heavily contaminated; 4 < *Igeo* ≤ 5, heavily to extremely contaminated; and *Igeo* > 5, extremely contaminated. The *Igeo* scores showed a good agreement with the *EF* results in terms of the degree of enrichment: the values for the various elements, on the whole, fall within the first two lowest *Igeo* categories, depicting a condition of limited contamination. The highest score, corresponding to “uncontaminated to moderately contaminated” sediments, was concerning Cd (1.3) for sample INT12 (a sample that also draws our attention in the discussion of *EF*s).

However, it is important to note that the *Igeo* scores evidenced more uncontaminated samples than *EF*. Indeed, several sediments, especially among the subtidal ones, that were classified as having “minor enrichments” according to *EF* are instead rated as uncontaminated by *Igeo*. This discordance between *Igeo* and *EF* classification was predictable, as already reported by many authors (e.g., Haris and Aris (2013); Loska et al. (2003); Nowrouzi and Pourkhabbaz (2014); Shafie et al. (2013); Sukri et al. (2018)). In fact, it is due to the normalization step implied in the *EF* formula, and particularly related to the choice of the reference element and the background matrix. Therefore, even considering that the normalization step of the *EF* was based on the local geochemical background values, the *Igeo* evaluation appears to be less reliable in the studied area.

#### Multielement contamination assessment results

**Fig. 6:**
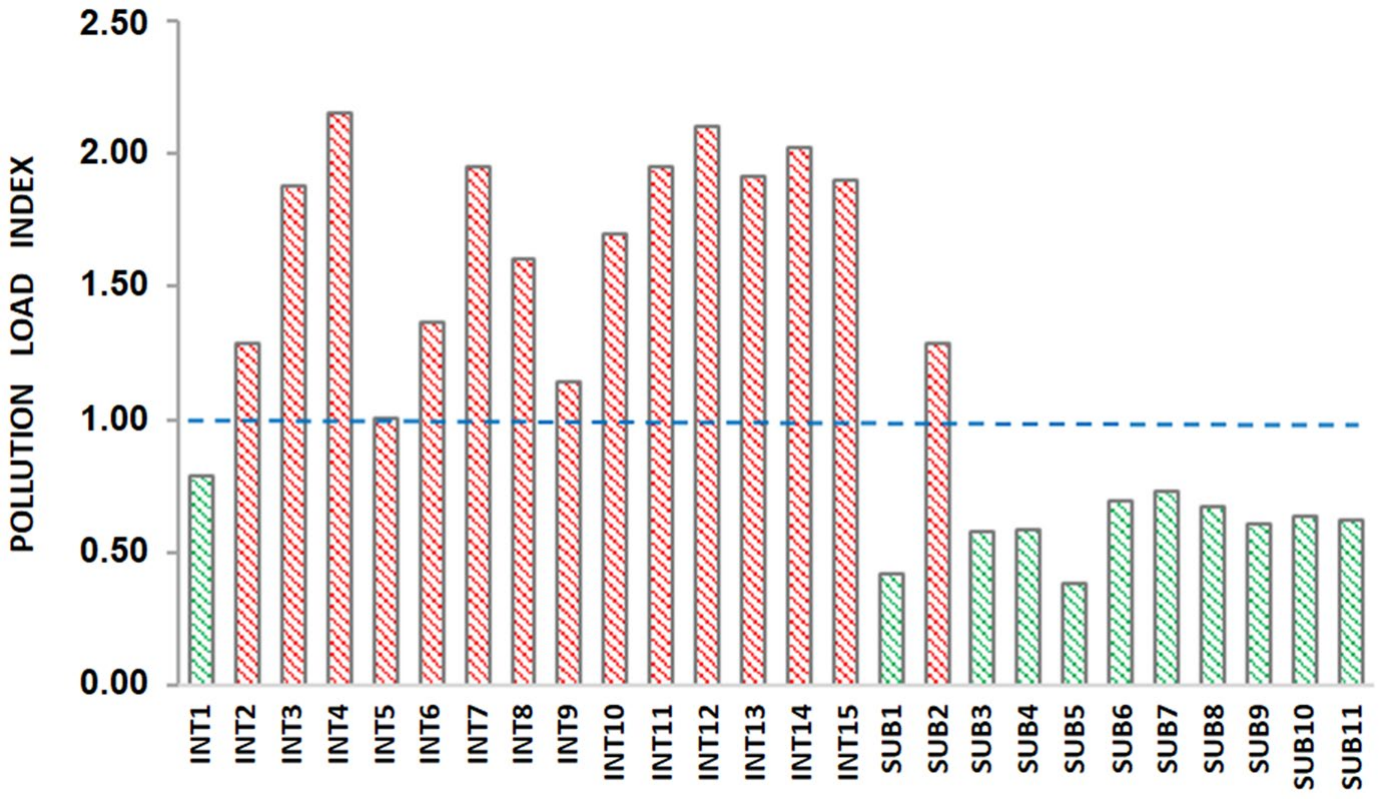
Pollution load index (*PLI*) of surface sediments from Khnifiss lagoon

The *PLI* results are shown in Fig. 6. Conversely to the previously discussed single element–based indices, *PLI* is multielement-based, accounting for the contribution of several heavy metals. In this case, *PLI* was calculated considering the five potentially most hazardous heavy metals (As, Cd, Cu, Ni, Pb) for the Khnifiss lagoon. According to Tomlinson et al. (1980), *PLI* > 1 values indicate sample stations that can be considered polluted, whereas if *PLI* < 1, there is a low level of metal pollution. All intertidal samples except INT1 showed a *PLI* greater than 1. The highest value was calculated for INT4 (*PLI* = 2.16). Thus, notwithstanding that from the previous indices the lagoon sediments are only characterized by minor enrichments for some elements, *PLI* also showed that from a general point of view, the intertidal samples are affected by pollutant inputs. Considering subtidal sediments, only the SUB2 sample exhibited a *PLI* greater than 1. Moreover, the differences between intertidal and subtidal sediments suggested that tidal cycles play a relevant role in pollutant transport and accumulation.

**Fig. 7:**
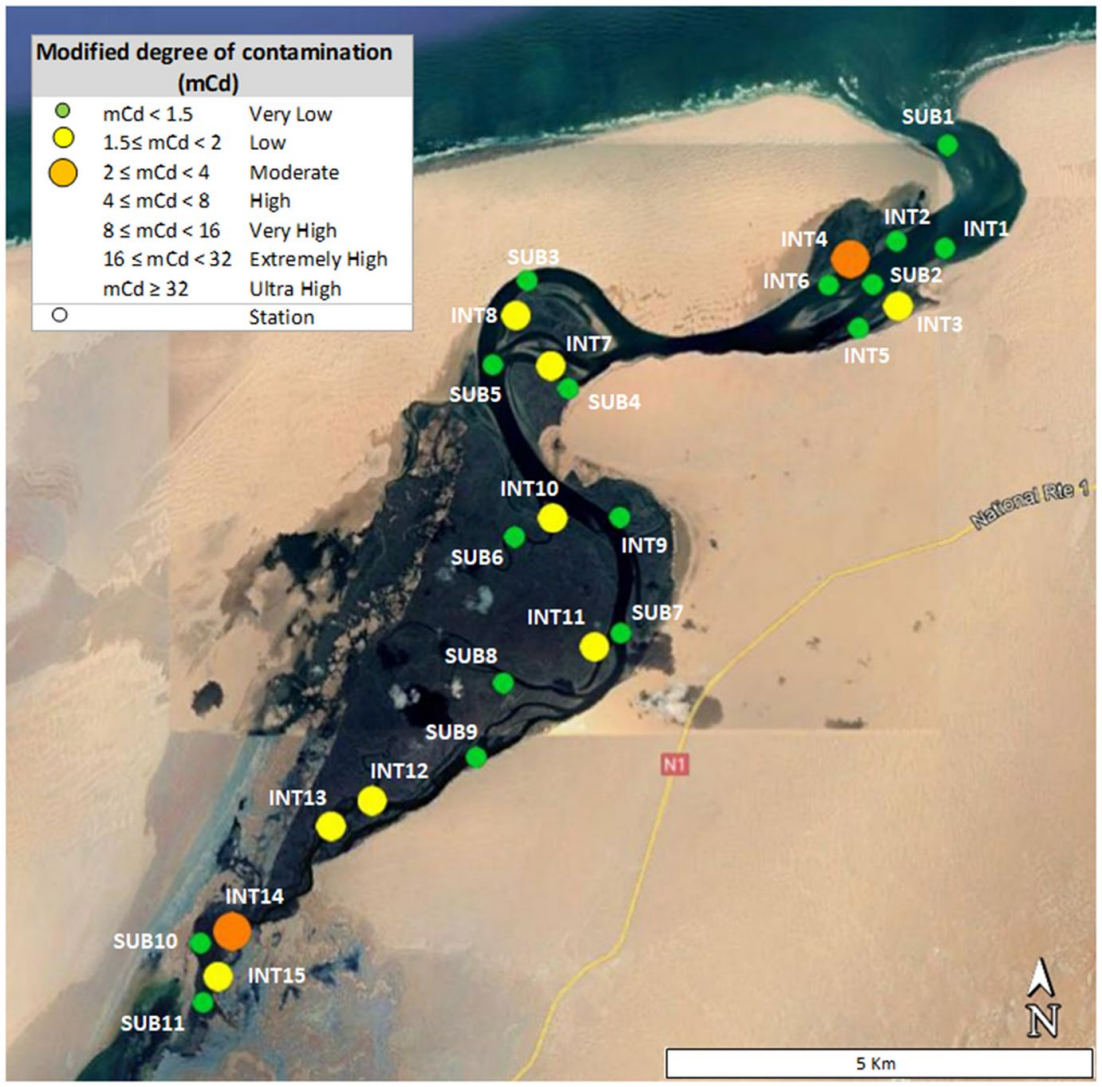
Map of modified degree of contamination (*mCd*) for heavy metals (As, Cd, Co, Cr, Cu, Mn, Ni, Pb, V, Zn) in the sediment of Khnifiss lagoon

Considering the surface sediment data, which represent the current heavy metal amount in the area, and *LGB*, indicating the natural geogenic occurrence of the elements, an attempt to display the overall spatial distribution of the heavy metal enrichments was made. A map based on *mCd* (Fig. 7) confirms again that only a very low or low heavy metal enrichment affects the lagoon. Furthermore, it also evidences that the contamination is distributed in the entire lagoon area but only in the intertidal sediments. Only two points showed moderate contamination, located in the lagoon’s mouth (sample INT4), where the tidal influence is stronger, and in the inner section (sample INT14), where the saltworks-related contribution is closer.

#### Potential ecological risk index (*RI*) results

According to the results of the contamination factor (*µCF*), which is the starting parameter for the calculation of ecological risk, the following sequence was highlighted (Table 6): Ni > Cu > V > As > Cr > Cd > Co = Pb > Zn > Mn. Intertidal samples were found to be more sensitive to contamination than subtidal samples (1 ≤ *µCF* < 3, corresponding to moderate contamination, and *µCF* < 1, corresponding to low contamination).

**Table 6:**
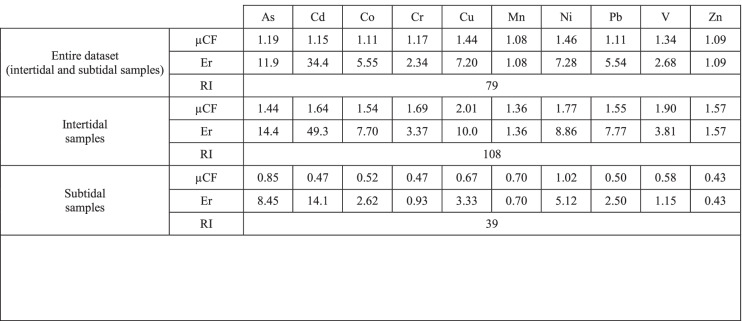
Values of single-element contamination factor (*µCF*) and corresponding potential ecological risk indices (*Er* and *RI*) for the Khnifiss lagoon

Classification according to Hakanson (1980): *µCF* < 1 low, 1 ≤ *CF* < 3 moderate, 3 ≤ *µCF* < 6 considerable, and *µCF* ≥ 6 very high. *Er* < 40 low, 40 ≤ *Er* < 80 moderate, 80 ≤ *Er* < 160 considerable, 160 ≤ *Er* < 320 high, and *Er* ≥ 320 very high. *RI* < 150 low, 150 ≤ *RI* < 300 moderate, 300 ≤ *RI* < 600 considerable, and *RI* ≥ 600 very high

The potential ecological risk posed by the investigated elements to the lagoon ecosystem is negligible (*RI* always less than 150). The risk factor (*Er*) associated with each individual heavy metal was classified as “low” (*Er* < 40), with the exception of Cd in intertidal sediments, which was rated as “moderate.” *Er* values followed the progression Cd > As > Ni > Cu > Co > Pb > V > Cr > Zn > Mn.

Table 7 reports the results of the potential ecological risk posed by Cd, Cr, Cu, Mn, Ni, Pb, and Zn, estimated for the five lagoons located along the Atlantic coast of Morocco (Khnifiss and other four sites). In order to calculate the risk indices, average surface sediment metal concentrations taken from the literature (Boutahar et al. (2019) and Maanan et al. (2015)) and background concentrations relative to each lagoon previously reported in Table 4 were used. As and Co were not considered in this elaboration, due to the absence of data in some lagoons.

**Table 7:** Individual potential ecological risk factor (*Er*) of Cd, Cr, Cu, Ni, Pb, and Zn and potential ecological risk index (*RI*) of five lagoons located along the coastline of Morocco

		Potential ecological risk index (*RI*)						
		Low		Moderate			Considerable	Very high
		Khnifiss (59)	Merja Zerga (42)	Sidi Moussa (200)	Nador (236)	Oualidia (279)		
Potential ecological risk factor (*Er*)	Low	Cd > Ni > Cu > Pb > Cr > Zn > Mn	Ni > Cu > Cr > Cd > Pb > Zn > Mn	Cu > Cr > Pb > Zn > Ni > Mn	Pb > Cu > Ni > Zn > Cr > Mn	Ni > Cr > Zn > Mn		
	Moderate					Pb > Cu		
	Considerable					Cd		
	High			Cd	Cd			
	Very high							

The results showed that Khnifiss and Merja Zerga lagoons can be associated with low risk (with slightly higher risk for Khnifiss), while Sidi Moussa, Nador, and Oualidia with moderate risk. In all four lagoons, the main contribution to ecological risk can be ascribed mainly to Cd. Only in the case of Merja Zerga did the factors show that Ni has greater relevance than Cd on the risk affecting the ecosystem.

## Conclusions

Surface sediments from the Khnifiss lagoon were analyzed to assess enrichment in heavy metals. The geochronological investigation of two sediment cores from the same area, dated by ^210^Pb and ^137^Cs, allowed to identify pre-industrial layers and ultimately to calculate site-specific background concentrations. The obtained data were used in this study as reference values in the calculation of an assortment of contamination indices, but also represent an important resource for future investigations focused on the time trend of the anthropogenic heavy metal enrichment in areas with similar geomorphological and geochemical characteristics.

The results showed that the area is affected by scarce anthropogenic impact, and only minor/moderated enrichments were detected. The sources of metals may be related to the various human activities in the proximity of the lagoon, in particular vehicular traffic associated with the adjacent National Route 1 (N1) highway, which runs alongside the Khnifiss National Park; motor fishing boats in the small harbors also located in the lagoon area; tourist vehicles circulating in the lagoon region; and machinery and trucks used in the extensive saltworks operating in the southern portion of the lagoon. Transport of contaminants related to nearby Tarfaya (the closest town to Khnifiss National Park) and the Canary Islands, and industrial emissions from the Atlantic coast of Morocco and western Algeria are also plausible contributors to enrichment. Intertidal sediments appeared to be more exposed to the heavy metal inputs, indicating a relevant role of tidal cycles in contaminants’ transport and deposition. According to the comparison with the other four Moroccan lagoons, Khnifiss showed a lower ecological risk.

In order to ensure proper environmental management of the Khnifiss lagoon, we suggest to periodically repeat the sampling campaign and to extend the investigation by including samples from areas not yet investigated.

## Supplementary Information

Below is the link to the electronic supplementary material.

Supplementary file1 (DOCX 19 KB)

Supplementary file2 (DOCX 19 KB)

## Data Availability

The datasets generated during and/or analyzed during the current study are available from the corresponding author on reasonable request.
