# Supplementary material for: Heavy metal content and potential ecological risk assessment of sediments from Khnifiss Lagoon National Park (Morocco)
Source: Environ Monit Assess. 2022 Apr 11;194(5):356. doi: 10.1007/s10661-022-10002-1 (PMC9001557; doi:10.1007/s10661-022-10002-1)
Supplement: Supplementary file 1 — Supplementary file1 (DOCX 19 KB) [file 10661_2022_10002_MOESM1_ESM.docx]

**Table ESM1:** Enrichment Factors (EFs) in surface sediments based on local background values

| Sample | As | Cd | Co | Cr | Cu | Mn | Ni | Pb | V | Zn | Mean |
| --- | --- | --- | --- | --- | --- | --- | --- | --- | --- | --- | --- |
| INT1 | 1.0 | 0.7 | **1.1** | 0.7 | 1.0 | **1.2** | **1.5** | 0.6 | 0.8 | 0.6 | 0.9 ± 0.3 |
| INT2 | 0.8 | 1.0 | **1.2** | 1.0 | **1.2** | 1.0 | **1.2** | 1.0 | 1.0 | 0.9 | 1.0 ± 0.1 |
| INT3 | 1.0 | **1.2** | **1.2** | **1.2** | **1.5** | **1.1** | **1.3** | **1.2** | **1.4** | **1.1** | 1.2 ± 0.2 |
| INT4 | 0.8 | **1.2** | **1.1** | **1.2** | **1.5** | 0.8 | **1.3** | **1.1** | **1.3** | **1.1** | 1.1 ± 0.2 |
| INT5 | **1.6** | 0.8 | **1.1** | **1.4** | **1.8** | **2.0** | **1.5** | **1.8** | **2.4** | **1.1** | 1.6 ± 0.5 |
| INT6 | 0.8 | **1.1** | 0.8 | 1.0 | **1.4** | 0.8 | **1.3** | 1.0 | 1.0 | 1.0 | 1.0 ± 0.2 |
| INT7 | 0.8 | 0.9 | **1.1** | **1.2** | **1.5** | 0.9 | **1.3** | **1.1** | **1.2** | **1.1** | 1.1 ± 0.2 |
| INT8 | 0.6 | 0.8 | 0.9 | 0.9 | **1.1** | 0.8 | 0.9 | **1.1** | 1.0 | **1.1** | 0.9 ± 0.2 |
| INT9 | 1.0 | 0.8 | 1.0 | 0.9 | **1.1** | 1.0 | 1.0 | 0.9 | **1.1** | 0.9 | 1.0 ± 0.1 |
| INT10 | 0.7 | **1.3** | 1.0 | **1.1** | **1.3** | 0.9 | **1.1** | **1.1** | **1.2** | 1.0 | 1.1 ± 0.2 |
| INT11 | **1.2** | **1.5** | **1.1** | **1.3** | **1.4** | **1.1** | **1.3** | 1.0 | **1.4** | **1.2** | 1.2 ± 0.2 |
| INT12 | **1.5** | **2.6** | **1.2** | **1.2** | **1.6** | 1.0 | **1.3** | 1.0 | **1.5** | **1.1** | 1.4 ± 0.5 |
| INT13 | 0.8 | 0.9 | 0.9 | **1.2** | **1.2** | 0.6 | **1.1** | 0.9 | **1.3** | 1.0 | 1.0 ± 0.2 |
| INT16 | **1.2** | 0.8 | 0.9 | **1.2** | **1.2** | 0.6 | 1.0 | 0.9 | **1.3** | **1.1** | 1.0 ± 0.2 |
| INT17 | 1.0 | 0.8 | **1.1** | **1.3** | **1.4** | 0.9 | **1.2** | **1.1** | **1.4** | **1.1** | 1.1 ± 0.2 |
| SUB1 | **1.4** | 0.5 | 0.4 | 0.4 | 0.5 | 0.7 | **1.3** | 0.6 | 0.4 | 0.4 | 0.7 ± 0.4 |
| SUB2 | 0.9 | 0.9 | **1.3** | 1.0 | **1.4** | **1.3** | **2.7** | 0.9 | **1.2** | 0.8 | 1.2 ± 0.6 |
| SUB3 | **1.8** | 0.8 | **1.4** | 0.7 | 0.7 | **1.3** | **3.0** | 0.8 | 0.8 | 0.5 | 1.2 ± 0.7 |
| SUB4 | **1.6** | 1.0 | 0.7 | 0.8 | 1.0 | **1.2** | **1.9** | 1.0 | **1.1** | 0.7 | 1.1 ± 0.4 |
| SUB5 | **2.3** | 0.9 | 0.6 | 0.5 | 0.6 | 0.9 | **2.1** | 0.8 | 0.7 | 0.5 | 1.0 ± 0.6 |
| SUB6 | **1.3** | 1.0 | 1.0 | 1.0 | **1.5** | **1.3** | **1.8** | **1.1** | **1.1** | 1.0 | 1.2 ± 0.3 |
| SUB7 | **1.1** | 0.6 | 0.7 | 0.7 | **1.1** | **1.1** | 1.0 | 0.7 | 1.0 | 0.7 | 0.9 ± 0.2 |
| SUB8 | **2.4** | **1.2** | **1.3** | **1.3** | **2.0** | **1.9** | **1.8** | **1.4** | **1.6** | **1.4** | 1.6 ± 0.4 |
| SUB9 | **1.3** | 0.6 | 0.6 | 0.7 | 0.9 | 1.0 | 0.9 | 0.7 | 0.8 | 0.6 | 0.8 ± 0.2 |
| SUB10 | 1.0 | 0.6 | 0.7 | 0.7 | 0.9 | **1.1** | 1.0 | 0.7 | 0.9 | 0.7 | 0.8 ± 0.2 |
| SUB11 | **1.2** | 0.6 | 0.6 | 0.7 | 1.0 | 1.0 | **1.1** | 0.7 | 0.7 | 0.6 | 0.8 ± 0.2 |
| Plain values: no enrichments  Bold values: minor enrichments | | | | | | | | | | | |
