# Supplementary material for: Heavy metal content and potential ecological risk assessment of sediments from Khnifiss Lagoon National Park (Morocco)
Source: Environ Monit Assess. 2022 Apr 11;194(5):356. doi: 10.1007/s10661-022-10002-1 (PMC9001557; doi:10.1007/s10661-022-10002-1)
Supplement: Supplementary file 2 — Supplementary file2 (DOCX 19 KB) [file 10661_2022_10002_MOESM2_ESM.docx]

**Table ESM2:** Geo- accumulation index (Igeo) in surface sediments, based on local background values

| Sample | As | Cd | Co | Cr | Cu | Mn | Ni | Pb | V | Zn | Mean |
| --- | --- | --- | --- | --- | --- | --- | --- | --- | --- | --- | --- |
| INT1 | - 0.7 | - 1.4 | - 0.6 | - 1.4 | - 0.8 | - 0.6 | - 0.2 | - 1.5 | - 1.1 | - 1.5 | - 1.0 ± 0.5 |
| INT2 | - 0.6 | - 0.2 | 0 | - 0.3 | 0 | - 0.2 | 0 | - 0.3 | - 0.2 | - 0.4 | - 0.2 ± 0.2 |
| INT3 | 0 | **0.3** | **0.3** | **0.4** | **0.6** | **0.1** | **0.4** | **0.3** | **0.5** | **0.2** | 0.3 ± 0.2 |
| INT4 | 0 | **0.6** | **0.4** | **0.6** | **0.9** | 0 | **0.7** | **0.5** | **0.7** | **0.5** | 0.5 ± 0.3 |
| INT5 | - 0.4 | - 1.4 | - 0.9 | - 0.6 | - 0.2 | - 0.1 | - 0.5 | - 0.3 | 0.2 | - 1.0 | - 0.5 ± 0.5 |
| INT6 | - 0.5 | - 0.2 | - 0.5 | - 0.3 | **0.2** | - 0.7 | **0.1** | - 0.2 | - 0.2 | - 0.3 | - 0.3 ± 0.3 |
| INT7 | - 0.1 | **0.2** | **0.4** | **0.5** | **0.8** | 0 | **0.6** | **0.4** | **0.6** | **0.4** | 0.4 ± 0.3 |
| INT8 | - 0.4 | - 0.1 | **0.1** | **0.1** | **0.5** | - 0.1 | **0.1** | **0.4** | **0.3** | **0.3** | 0.1 ± 0.3 |
| INT9 | - 0.3 | - 0.7 | - 0.3 | - 0.4 | - 0.2 | - 0.3 | - 0.3 | - 0.5 | - 0.2 | - 0.5 | - 0.4 ± 0.1 |
| INT10 | - 0.3 | **0.4** | **0.1** | **0.2** | **0.5** | - 0.1 | **0.2** | **0.2** | **0.3** | **0.1** | 0.2 ± 0.2 |
| INT11 | **0.3** | **0.6** | **0.2** | **0.4** | **0.5** | **0.1** | **0.4** | **0.1** | **0.5** | **0.3** | 0.3 ± 0.2 |
| INT12 | **0.5** | **1.3** | **0.1** | **0.2** | **0.5** | - 0.1 | **0.3** | - 0.1 | **0.5** | **0.1** | 0.3 ± 0.4 |
| INT13 | 0 | **0.2** | **0.2** | **0.6** | **0.7** | - 0.2 | **0.5** | **0.3** | **0.8** | **0.5** | 0.4 ± 0.3 |
| INT14 | **0.7** | **0.1** | **0.2** | **0.6** | **0.7** | - 0.3 | **0.4** | **0.2** | **0.8** | **0.5** | 0.4 ± 0.3 |
| INT15 | **0.3** | 0 | **0.3** | **0.6** | **0.7** | 0 | **0.5** | **0.3** | **0.7** | **0.4** | 0.4 ± 0.3 |
| SUB1 | - 1.0 | - 2.4 | - 2.8 | - 2.9 | - 2.4 | - 2.1 | - 1.1 | - 2.1 | - 2.7 | - 3.0 | - 2.3 ± 0.7 |
| SUB2 | - 0.6 | - 0.7 | - 0.2 | - 0.6 | 0 | - 0.2 | **0.9** | - 0.7 | - 0.3 | - 0.8 | - 0.3 ± 0.5 |
| SUB3 | - 0.8 | - 2.0 | - 1.2 | - 2.2 | - 2.1 | - 1.2 | 0 | - 2.0 | - 1.9 | - 2.6 | - 1.6 ± 0.8 |
| SUB4 | - 1.0 | - 1.7 | - 2.1 | - 2.0 | - 1.7 | - 1.4 | - 0.8 | - 1.6 | - 1.6 | - 2.3 | - 1.6 ± 0.5 |
| SUB5 | - 1.0 | - 2.4 | - 2.9 | - 3.1 | - 2.8 | - 2.4 | - 1.1 | - 2.6 | - 2.8 | - 3.1 | - 2.4 ± 0.8 |
| SUB6 | - 1.1 | - 1.5 | - 1.5 | - 1.5 | - 0.9 | - 1.2 | - 0.7 | - 1.4 | - 1.3 | - 1.5 | - 1.3 ± 0.3 |
| SUB7 | - 0.7 | - 1.7 | - 1.3 | - 1.4 | - 0.7 | - 0.7 | - 0.8 | - 1.4 | - 0.9 | - 1.4 | - 1.1 ± 0.4 |
| SUB8 | - 0.7 | - 1.7 | - 1.6 | - 1.5 | - 0.9 | - 1.0 | - 1.1 | - 1.5 | - 1.3 | - 1.5 | - 1.3 ± 0.3 |
| SUB9 | - 0.6 | - 1.8 | - 1.7 | - 1.6 | - 1.2 | - 1.0 | - 1.2 | - 1.6 | - 1.4 | - 1.8 | - 1.4 ± 0.4 |
| SUB10 | - 0.9 | - 1.7 | - 1.5 | - 1.5 | - 1.1 | - 0.9 | - 1.0 | - 1.6 | - 1.2 | - 1.6 | - 1.3 ± 0.3 |
| SUB11 | - 0.8 | - 1.8 | - 1.8 | - 1.7 | - 1.1 | - 1.1 | - 1.0 | - 1.7 | - 1.6 | - 1.9 | - 1.5 ± 0.4 |
| Plain values: uncontaminated  Bold values: uncontaminated to moderately contaminated | | | | | | | | | | | |
